# Large retroperitoneal lymphadenopathy and increased risk of venous thromboembolism in patients receiving first‐line chemotherapy for metastatic germ cell tumors: A study by the global germ cell cancer group (G3)

**DOI:** 10.1002/cam4.2674

**Published:** 2019-11-12

**Authors:** Ben Tran, Jose M. Ruiz‐Morales, Enrique Gonzalez‐Billalabeitia, Anna Patrikidou, Eitan Amir, Christoph Seidel, Carsten Bokemeyer, Christian Fankhauser, Thomas Hermanns, Alexey Rumyantsev, Alexey Tryakin, Margarida Brito, Aude Fléchon, Edmond Michael Kwan, Tina Cheng, Daniel Castellano, Xavier Garcia del Muro, Anis A. Hamid, Margaret Ottaviano, Giovannella Palmieri, Robert Kitson, Alison Reid, Daniel Y. C. Heng, Philippe L. Bedard

**Affiliations:** ^1^ Department of Medical Oncology Peter MacCallum Cancer Centre Melbourne Vic. Australia; ^2^ Tom Baker Cancer Centre Calgary AB Canada; ^3^ Hospital Universitario Morales Meseguer – IMIB UCAM Murcia Spain; ^4^ Royal Marsden Hospital London UK; ^5^ Division of Medical Oncology and Hematology Department of Medicine Princess Margaret Cancer Centre University of Toronto Toronto ON Canada; ^6^ Department of Oncology, Hematology, BMT with Section Pneumology Hubertus Wald Tumorzentrum University Medical Centre Hamburg‐Eppendorf Hamburg Germany; ^7^ University Hospital Zurich University of Zurich Zurich Switzerland; ^8^ NN Blokhin Russian Cancer Research Centre and Research Institute of Oncology at BSMU Moskva Russia; ^9^ Instituto Português de Oncologia de Lisboa Francisco Gentil Lisbon Portugal; ^10^ Centre Léon Bérard Lyon France; ^11^ Hospital Universitario 12 de Octubre Madrid Spain; ^12^ Institut Catala d'Oncologia Idibell University of Barcelona Barcelona Spain; ^13^ Olivia Newton John Cancer Wellness and Research Centre Heidelberg Vic. Australia; ^14^ CRTR Rare Tumors Reference Center Università Degli Studi di Napoli Federico II Napoli Italy

**Keywords:** deep vein thrombosis, germ cell tumor, pulmonary embolism, testicular cancer, vascular access device, venous thromboembolism

## Abstract

**Background:**

Metastatic germ cell tumor (mGCT) patients receiving chemotherapy have increased risk of life‐threatening venous thromboembolism (VTE). Identifying VTE risk factors may guide thromboprophylaxis in this highly curable population.

**Methods:**

Data were collected from mGCT patients receiving first‐line platinum‐based chemotherapy at 22 centers. Predefined variables included International Germ Cell Cancer Collaborative Group (IGCCCG) risk classification, long‐axis diameter of largest retroperitoneal lymph node (RPLN), Khorana score, and use of indwelling vascular access device (VAD). VTE occurring at baseline, during chemotherapy and within 90 days, was analyzed.

**Results:**

Data from 1135 patients were collected. Median age was 31 years (range 10‐74). IGCCCG risk was 64% good, 20% intermediate, and 16% poor. VTE occurred in 150 (13%) patients. RPLN >3.5 cm demonstrated highest discriminatory accuracy for VTE (AUC 0.632, *P* < .001) and was associated with significantly higher risk of VTE in univariable analysis (22% vs 8%, OR 3.0, *P* < .001) and multivariable analysis (OR 1.8, *P* = .02). Other significant risk factors included, Khorana score ≥3 (OR 2.6, *P* = .008) and VAD use (OR 2.7, *P* < .001).

**Conclusions:**

Large RPLN and VAD use are independent risk factors for VTE in mGCT patients receiving chemotherapy. VAD use should be minimized in this population and thromboprophylaxis might be considered for large RPLN.

## Graphical abstract

Venous thromboembolism can cause morbidity in germ cell tumor patients receiving chemotherapy; large retroperitoneal lymphadenopathy (RPLN) and indwelling vascular access devices (VAD) are significant VTE risk factors. VAD insertion should be avoided and thromboprophylaxis can be considered for large RPLN.

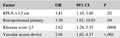

## 1 INTRODUCTION

Germ cell tumors (GCTs) represent the most common solid cancer affecting young men.^1^ Even in the metastatic setting, GCT are highly curable, due to their extraordinary responsiveness to cisplatin‐based chemotherapy.^1^ Subsequently, much focus is now placed on survivorship, and minimizing treatment‐related toxicities while maintaining cure in these young men.

The increased risk of venous thromboembolism (VTE) in metastatic germ cell tumor (mGCT) patients is well recognized.^2^, ^3^, ^4^, ^5^, ^6^, ^7^, ^8^ While cisplatin‐based chemotherapy is highly curative, it may increase VTE risk,^9^ with an incidence of approximately 10% in mGCT patients.^3^, ^4^, ^5^, ^6^, ^7^ VTE can cause significant morbidity and even mortality.^10^ Hemorrhagic complications may occur in mGCT, due to treatment‐induced thrombocytopenia and the presence of choriocarcinoma.^11^ There is a need to determine predictors of VTE risk in mGCT patients, in order to identify patients most likely to benefit from thromboprophylaxis.

Previously, in a small cohort, we reported that patients with large retroperitoneal lymph node (RPLN) metastases (>5 cm) were at significantly higher risk of VTE compared to those without (22% vs 5%, OR 5.26, *P* = .001). A similar but nonsignificant effect was observed in an underpowered validation cohort (14.2% vs 6%, OR 2.54, *P* = .16).^3^

The Global Germ Cell Collaborative Group (G3) is an international consortium committed to the management of GCT. This G3 study aimed to validate large RPLN metastases as an independent risk factor for VTE in mGCT patients receiving first‐line platinum‐based chemotherapy. Secondary objectives aimed to determine the optimal cutoff for RPLN size as a predictor of VTE risk, to evaluate other potential risk factors, to assess the impact of VTE on overall survival (OS), and to explore the safety and benefit of thromboprophylaxis.

## 2 METHODS

The study protocol was circulated through the G3 group for expression of interest to participate. Institutional research ethics board approval was obtained.

### 2.1 Study population

The cohort consists of men diagnosed with mGCT (Stage 1S, 2, or 3 by American Joint Committee on Cancer [AJCC] stage classification^12^) treated with first‐line platinum‐based chemotherapy with curative intent between 1 January 2000 and 31 December 2014. Patients with prior chemotherapy for GCT or history of secondary malignancy were excluded. Consecutive patients were enrolled from each site. Patients from the original study were not included.^3^

### 2.2 Data collection

Retrospectively collected data were entered into case report forms. Baseline (pre‐chemotherapy) variables included age, presence of an indwelling vascular access device (VAD), hospitalization (for any reason), primary tumor site, histology, AJCC stage, International Germ Cell Cancer Collaborative Group (IGCCCG) risk classification, and RPLN size. Baseline laboratory investigations were also recorded, including hemoglobin, leukocyte count, and platelet count. Khorana score ≥3, a known VTE risk factor in cancer patients, was calculated.^2^ Renal impairment was defined as creatinine clearance (CrCl) <75 mL/min (calculated using the Cockcroft Gault formula) at any point during chemotherapy as this has previously been associated with increased VTE risk.^13^ VTE was defined as either deep vein thrombosis (DVT) or pulmonary embolism (PE). Both symptomatic VTE and asymptomatic VTE detected incidentally during routine imaging were included in this analysis. Superficial venous thrombosis or thrombophlebitis were not considered VTE events. VTE events must have occurred between diagnosis of metastatic disease and 90 days following chemotherapy completion. Where available, long‐axis diameter measured in axial cross section (selected given its use in AJCC staging) of the largest RPLN metastasis from the pre‐chemotherapy CT scan was recorded. If unavailable, we used imaging reports to determine if the maximal diameter measured was >5 cm or ≤5 cm. Use of thromboprophylaxis and bleeding events were recorded. Last follow‐up date and survival status were collected.

### 2.3 Statistical considerations

Categorical variables were compared between cohorts using Fisher's exact test and Kruskal‐Wallis test for two or greater than two variables, respectively. Continuous variables were compared using the Mann‐Whitney *U* test. Optimal cutoff for RPLN size in defining VTE risk was determined using the area under receiver operator characteristic curve (AUROC) and quantified as the previously described c‐statistic.^14^ Risk factors associated with VTE were evaluated using univariable logistic regression. Multivariable analysis was performed using manual variable reduction; all variables with *P* < .1 in univariable analysis were included in the multivariable model individually, in addition to any variable showing evidence of confounding as previously described.^15^ Patients who received prolonged thromboprophylaxis (≥7 days) were excluded from these analyses; patients receiving short‐term thromboprophylaxis (<7 days) were included. An exploratory stratified analysis assessing large RPLN and VAD use was conducted using univariable logistic regression. Overall survival (OS) was assessed using the Cox proportional hazards method; VTE was classified as a time‐dependent covariable. Statistical analyses were performed using SPSS version 21 (IBM Corp). The threshold for statistical significance was a two‐sided *P* < .05 with no adjustment for multiple testing.

## 3 RESULTS

We identified 1135 patients with mGCT treated with first‐line platinum‐based chemotherapy across 22 sites in 10 countries. Table 1 describes the patient characteristics. VTE events occurred in 150 (10%) patients. Of the potential VTE risk factors, Khorana score ≥3 was infrequent (7%), 33% of patients had baseline lactate dehydrogenase (LDH) >1.5× upper limit of normal (ULN), and 19% used VAD. The maximal long‐axis diameter of the largest RPLN was available in 1011 (89%) patients, and the median diameter in this group was 3.0 cm, 25% had RPLN > 5 cm and 38% had RPLN >3.5 cm.

**Table 1:** Patient characteristics of the entire cohort (N = 1135)

Country	
Canada	276 (24%)
Spain	260 (23%)
UK	152 (13%)
Switzerland	96 (8%)
Italy	77 (7%)
Germany	74 (7%)
Russia	69 (6%)
Australia	53 (5%)
Portugal	49 (4%)
France	29 (3%)
Age at diagnosis	
Median (range)	31.1 (10.5‐74.0)
Primary site	
Testis	1 046 (92%)
Retroperitoneal	19 (2%)
Mediastinal	33 (3%)
Other	36 (3%)
Unknown	1 (<1%)
Histology	
Seminoma	308 (27%)
Non‐seminoma/Mixed	821 (72%)
Unknown	6 (1%)
AJCC stage (at time of chemotherapy)	
1S	61 (5%)
2^a^	7 (1%)
2A	194 (17%)
2B	162 (14%)
2C	111 (10%)
3	578 (51%)
Unknown	22 (2%)
IGCCCG risk classification	
Good	727 (64%)
Intermediate	224 (20%)
Poor	182 (16%)
Unknown	2 (<1%)
Chemotherapy regimen^b^	
BEP	931 (82%)
EP	88 (8%)
VIP	26 (2%)
TIP	3 (<1%)
Other	86 (8%)
Body surface area^c^	
Median	1.97
BSA >2	461 (44%)
Body mass index^c^	
Median	25.2
BMI >35	37 (4%)
Khorana score	
1	714 (63%)
2	230 (20%)
3	71 (6%)
4	9 (1%)
Unknown	111 (10%)
Smoking status	
Nonsmoker	538 (47%)
Current‐smoker	324 (29%)
Ex‐smoker	111 (10%)
Unknown	162 (14%)
Vascular access device inserted	
Yes	218 (19%)
No	640 (56%)
Unknown	277 (24%)
Renal function during chemotherapy^d^	
Median GFR (range)	120 mL/min (9‐309)
GFR <75 mL/min	64 (8%)
Hospitalizations	
Yes	491 (43%)
No	367 (32%)
Unknown	277 (25%)
Coagulopathy or known past history VTE	
Coagulopathy/VTE	14 (1%)
LDH	
<1.5× ULN	735 (65%)
1.5‐5× ULN	270 (24%)
5‐10× ULN	58 (5%)
>10× ULN	40 (4%)
Unknown	32 (2%)
Retroperitoneal lymph node size	
Median size^e^	3.0 cm
RPLN >5 cm	288 (25%)
RPLN ≤5 cm	846 (74%)
Unknown	1 (1%)
RPLN >3.5 cm	431 (38%)
RPLN ≤3.5 cm	580 (52%)
Unknown	124 (10%)
Prolonged prophylactic anticoagulation >7 days	
Yes	81 (7%)
No	1 035 (91%)
Unknown	19 (2%)
Venous thromboembolism (VTE)	
Occurrence of VTE	150 (10%)

a Identified as stage 2 but further sub‐classification unavailable.

b BEP = Bleomycin, Etoposide, Cisplatin; BSA = Body Surface Area; EP = Etoposide, Cisplatin; VIP = Etoposide, Ifosfamide, Cisplatin; TIP = Paclitaxel, Ifosfamide, Cisplatin.

c Available for 1051 (93%) of patients.

d Available for 764 (70%) of patients.

e Based upon for 1011 (89%) of patients with actual measurement recorded.

Table 2 details the VTE events, most occurred during or within 90 days of chemotherapy (64%). Intrabdominal DVT was the most common location (30%). Most VTE were symptomatic (55%) and less than half required hospitalization (41%). There was one VTE‐related death.

**Table 2:** VTE characteristics

Total number of VTE	150
Timing of VTE diagnosis	
Immediately prior to chemotherapy initiation	52 (35%)
During chemotherapy	78 (52%)
Immediately following chemotherapy completion	18 (12%)
Post‐chemotherapy, Postoperative setting	2 (1%)
Location of VTE	
Abdominal DVT (incl. IVC, iliac veins)	45 (30%)
Upper limb DVT (incl. subclavian, brachial veins)	11 (7%)
Lower limb DVT (incl. femoral vein)	27 (18%)
Pulmonary Embolus	42 (28%)
Other	4 (3%)
Vascular Access Device associated	21 (14%)
Presentation of VTE	
Symptomatic	83 (55%)
Incidental on imaging	39 (26%)
Unknown	28 (19%)
Complications of VTE	
Death due to VTE	1 (1%)
Hospitalization due to VTE	62 (41%)

In the subset with available long‐axis diameter, RPLN was associated with increased VTE risk when assessed as a continuous variable (OR 1.48, 95% CI 1.00, 2.18, *P* = .05). AUROC analysis determined that RPLN >3.5 cm had greater discriminatory accuracy (AUC 0.632, *P* < .0001) for VTE than RPLN > 5 cm (Figure S1A). Subsequently, 3.5 cm was selected as the ideal cutoff for statistical analyses.

Table 3 describes results of the univariable analysis of VTE risk factors. RPLN >3.5 cm was a statistically significant risk factor; VTE occurred in 22% of RPLN >3.5 cm compared to 8% of RPLN ≤3.5. Other significant risk factors included IGCCCG poor risk, LDH >5× ULN, retroperitoneal primary, Khorana score ≥3, CrCl <75 mL/min, and VAD use. Hospital admission and BSA were not significant risk factors. Multivariable analysis confirmed large RPLN >3.5 cm, retroperitoneal primary, Khorana score ≥3, and VAD as independent significant risk factors for VTE (Table 4). A sensitivity analysis was performed, excluding patients with VTE diagnosed between mGCT diagnosis and the start of chemotherapy. This demonstrated that both RPLN >3.5 cm (OR 2.19, 95% CI 1.36‐3.54, *P* = .001) and VAD (OR 4.93 95% 2.90‐8.37, *P* < .001) remained significantly associated with VTE.

**Table 3:** Risk factors for VTE (univariable analysis)

Factor	No. of patients N = 1 054^a^	No. of VTE (%) N = 138 (13%)	OR	95% CI	*P*
RPLN 3.5 cm cutoff^b^					
RPLN ≤3.5 cm	931	42 (8%)	—	—	—
RPLN >3.5 cm	378	82 (22%)	2.98	2.04, 4.34	<.001
RPLN 5 cm cutoff^c^					
RPLN ≤5 cm	810	84 (10%)	—	—	—
RPLN >5 cm	243	54 (22%)	2.6	1.77, 3.81	<.001
Primary site^d^					
Testicular primary	979	118 (12%)	—	—	—
Mediastinal primary	27	6 (22%)	2.16	0.78, 5.99	.14
Retroperitoneal primary	15	6 (40%)	5.76	1.97, 16.82	.001
IGCCCG Risk Classification^e^					
Good prognosis	693	64 (9%)	—	—	—
Intermediate prognosis	204	31 (15%)	1.72	1.08, 2.74	.02
Poor prognosis	155	43 (28%)	3.26	2.06, 5.17	<.001
LDH categories^f^					
<1.5× ULN	702	61 (9%)	—	—	—
1.5‐ 5× ULN	244	50 (20%)	2.55	1.68, 2.74	<.001
5‐10× ULN	48	13 (27%)	3.61	1.78, 7.32	<.001
>10× ULN	30	9 (30%)	4.68	1.95, 11.22	.001
LDH cutoff^f^					
<5× ULN	946	111 (12%)	—	—	—
>5× ULN	78	22 (28%)	2.92	1.70‐5.02	<.001
Khorana score^g^					
0‐2	882	105 (12%)	—	—	—
>3	66	20 (30%)	3.19	1.80, 5.64	<.001
Hospitalization^h^					
No	359	40 (11%)	—	—	—
Yes	413	64 (15%)	1.44	0.94‐2.19	.1
BSA^i^					
<2	621	83 (13%)	—	—	—
>2	414	52 (13%)	0.9	0.62‐1.31	.58
Vascular access device^j^					
No	602	58 (10%)		—	—
Yes	177	51 (29%)	3.46	2.22, 5.40	<.001
Renal function^k^					
CrCl >75 mL/min	668	89 (13%)	—	—	—
CrCl <75 mL/min	51	16 (31%)	3.48	1.87, 6.49	<.001

a Excludes patients who received prophylactic anticoagulation.

b Unknown in 123 patients.

c Unknown in 1 patient.

d Unknown or other site in 33 patients.

e Unknown in 2 patients.

f Unknown in 30 patients.

g Unknown in 106 patients.

h Unknown in 277 patients.

i Unknown in 81 patients.

j Unknown in 275 patients.

k Unknown in 335 patients.

**Table 4:** Statistically significant risk factors for VTE (multivariable analysis)^a^

Factor	OR	95% CI	*P*
RPLN >3.5 cm	1.81	1.10, 3.00	.02
Retroperitoneal primary	3.30	1.01, 10.83	.04
Khorana score ≥3	2.62	1.28, 5.35	.0008
Vascular access device	2.66	1.62, 4.37	<.001

a Excludes patients who received prophylactic anticoagulation.

An exploratory stratified analysis for RPLN and VAD was conducted. In patients without VAD (n = 559), RPLN >3.5 cm was strongly associated with VTE (OR 4.15, 95% CI 2.16‐7.99, *P* < .001), whereas in patients with VAD (n = 218) the association was nonsignificant (OR 1.61 95% CI 0.80‐3.21, *P* = .18). The test for interaction between VAD and RPLN >3.5 cm was statistically significant (*P* = .04), suggesting that in those with VAD, RPLN >3.5 cm has a lesser effect on VTE risk.

There were 81 patients who received prolonged thromboprophylaxis, mostly low molecular weight heparin (LMWH) (n = 80). The VTE incidence in this thromboprophylaxis group was 15% (12 of 81) compared to 13% (138 of 1,054) in the non‐thromboprophylaxis group. VTE risk factors were more common in the thromboprophylaxis patients (RPLN >3.5 cm 65% vs 36%, *P* < .001; VAD use 50% vs 17%, *P* < .001). Median duration of thromboprophylaxis was 45 days (range 8‐239), while the median duration of chemotherapy was 65 days (range 33‐184). An additional 48 patients received short‐term (<7 days) prophylaxis prescribed for inpatient hospital admissions.

In the entire cohort, there were 17 documented bleeding events (1.5%), resulting in nine hospitalizations, with eight requiring intervention (Table S1B). There were two bleeding‐related deaths, neither were receiving anticoagulation. There were no bleeding events identified in those receiving thromboprophylaxis. Two bleeding events occurred in the 140 patients who received therapeutic anticoagulation for VTE (1.4%).

After a median follow‐up of 49 months, there were 95 deaths. The 2‐year OS was 99% for good, 95% for intermediate, and 71% for poor IGCCCG prognosis categories. In univariable OS analysis, VTE, RPLN >3.5 cm, and IGCCCG risk group were significant prognostic factors, while in multivariable analysis, only IGCCCG risk group remained significant (Table S1C).

## 4 DISCUSSION

This multinational G3 consortium study was designed to validate large RPLN as a VTE risk factor in mGCT patients receiving chemotherapy. We found that large RPLN independently predicted risk of VTE, and a cutoff of 3.5 cm allowed for greater discriminatory accuracy and superior predictive accuracy compared to our original cutoff of 5 cm.^3^ Our data support large RPLN as a relevant biomarker that should be considered when assessing VTE risk in this patient population.

Several publications have identified large RPLN as a risk factor for VTE in mGCT patients.^3^, ^4^, ^5^, ^6^ Gizzi et al^6^ reported that enlarged RPLN was significantly associated with VTE in multivariable analyses. Similarly, a single‐institution Austrian study found RPLN >5 cm was a strong predictor of VTE (stratified HR 3.29, *P* = .002).^5^ Our study is the largest cohort to date of chemotherapy‐treated mGCT analyzed for VTE and confirms large RPLN as a significant risk factor.

We also identified VAD use as a significant VTE risk factor. In our cohort, 19% of patients utilized VADs, with 29% subsequently developing VTE. Prior studies, hindered by heterogeneous cohorts and small sample sizes, estimated the incidence of VAD‐associated VTE in mGCT patients to be 8%‐59%.^7^, ^16^, ^17^, ^18^ The increased VTE risk associated with VAD use in our study suggests that routine insertion of VADs to facilitate chemotherapy administration should be avoided. In situations where VAD use is necessary (eg, poor venous access, patient preference), thromboprophylaxis could be considered as a means of reducing VAD‐associated VTE.

Other significant VTE risk factors were also identified. Khorana score ≥3, consistent with previous reports, was associated with increased risk;^2^, ^3^ however, it only captured 20 (16%) VTE events, restricting its utility in this patient group. Renal impairment, defined by CrCl <75 mL/min, was also associated with increased risk; however, as it is logged at any point during chemotherapy (and not at baseline), its utility as a predictive risk factor is limited. Only 15 patients had an extragonadal retroperitoneal primary tumor and its role as a VTE risk factor should be interpreted with caution. Unexpectedly, hospitalizations were more frequent than expected (43%) but not a significant VTE risk factor. Some participating centers regularly administered chemotherapy regimens such as bleomycin (BEP) as an inpatient and given these patients are not acutely unwell, they are unlikely to harbor the same VTE risks as other hospitalized patients.

A recent study by the Spanish Germ Cell Group suggested that the occurrence of VTE in mGCT patients was associated with poorer progression‐free survival (PFS; HR 2.29, *P* = .02) and OS (HR 5.14, *P* < .001).^4^ While Bezan et al reported a fourfold increased risk of death with onset of VTE (HR 4.0, *P* = .03), this association did not persist in multivariable analyses after adjusting for tumor stage.^5^ In our study, VTE was associated with significantly poorer OS in univariable analysis (2‐year OS 85% vs 95%, HR 2.84, *P* < .001), but this was not confirmed in multivariable analysis (HR 1.51, *P* = .10). As such, we were unable to validate VTE as an independent risk factor for OS.

While our study confirmed that large RPLN metastases predict for higher risk of VTE in mGCT, the role of thromboprophylaxis in these patients is unproven. One retrospective study demonstrated a twofold reduction in VTE with thromboprophylaxis, yet subsequent multivariable analyses using a matching model failed to show statistical significance (OR 0.50, *P* = .09).^6^ Others have found no difference in VTE events with this approach.^19^ Given the retrospective nature of our study and the selection bias involved in analyzing patients who received thromboprophylaxis, we cannot comment on the safety nor efficacy of thromboprophylaxis in mGCT patients.

Ideally, a prospective, randomized clinical trial would be conducted to demonstrate the benefit of thromboprophylaxis in mGCT with RPLN >3.5 cm. Currently, ASCO guidelines recommend against routine thromboprophylaxis for cancer outpatients receiving chemotherapy,^20^ with the only exception being multiple myeloma where the risk of VTE is estimated to be 12%‐36% when receiving thalidomide and dexamethasone.^21^ Our data demonstrate the risk of VTE in mGCT patients with RPLN >3.5 cm (22%) to be comparable, and subsequently, thromboprophylaxis may have a role in these patients. While some centers have already adopted routine thromboprophylaxis in this high‐risk patient population,^6^, ^22^ given the risks of catastrophic bleeding in this curable population, a trial is required to determine if this is in our patients' best interest.

Our study has several limitations. Missing data were evident for multiple risk factors; however, our large sample size provided adequate power to overcome this. Our study did not include VTE events occurring beyond 90 days of completing chemotherapy, despite some data suggesting VTE risk persists beyond this period.^6^, ^7^ We included patients receiving short‐term thromboprophylaxis in VTE risk analyses, pragmatically defining a cutoff of 7 days, as we did not believe this short duration would significantly impact VTE risk. Maximal RPLN diameter was assessed by individual investigators rather than by central review, although interobserver variability was minimized through the provision of training materials to each site. Finally, as with any retrospective study, there is significant heterogeneity in practice patterns.

In conclusion, our large multinational study examining risk factors for VTE in mGCT patients receiving first‐line platinum‐based chemotherapy confirmed large RPLN as an independent risk factor for VTE and identified 3.5 cm as the optimal cutoff to identify patients who may benefit from thromboprophylaxis. We have also confirmed VAD use as a significant risk factor for VTE. Our findings have important clinical implications. Routine VAD insertion should be avoided in mGCT patients receiving chemotherapy, and thromboprophylaxis may have a role for patients with RPLN >3.5 cm and those where use of VAD is necessary, although prospective trials are desperately needed.

## CONFLICT OF INTEREST

Ben Tran: Consulting and Honraria from Amgen, Astellas, Bayer, Bristol‐Myers Squibb, Janssen‐Cilag, Sanofi, Novartis, Ipsen; Research funding from Amgen, Astellas, Janssen‐Cilag, Pfizer, Bristol‐Myers Squibb, Astra Zeneca, Ipsen. Jose M Ruiz‐Morales: Consulting from Novartis, Asofarma, Bristol‐Myers Squibb; Speaker's bureau from Asofarma and Bristol‐Myers Squibb. Enrique Gonzalez‐Billalabeitia: Travel from Bristol‐Myers Squibb, Pfizer, Janssen and Astellas. Anna Patrikidou: Honoraria/Consulting from Bristol‐Myers Squibb, Bayer, Astellas. Eitan Amir: Honoraria from Apobiologix, Agendia, Myriad Genetics; Expert Testimony for Genetech/Roche. Carsten Bokemeyer: Research grant from Sanofi, Honoraria/Consulting: Sanofi, Roche; Lilly; Merck Sharp and Dohme; Bristol‐Myers Squibb; Merck Darmstadt; Travel from Merck Serono, Sanofi, Pfizer, Bristol‐Myers Squibb. Alexey Tryakin: Speakers Bureau and Travel from Merck, Sanofi, Bayer, Lilly, Biocad, Veropharm, Roche. Margarida Brito: Travel from Roche and Bristol‐Myers Squibb. Daniel Castellano: Honoraria from Pfizer, Roche, Astellas, Novartis, Janssen; Consulting from Astra Zeneca, Roche, Pfizer, Janssen. Xavier Garcia del Muro: Consulting or Advisory Role ‐ Bristol‐Myers Squibb; Ipsen; Lilly; Pfizer; PharmaMar; Roche. Speakers' Bureau – Pfizer. Travel, Accommodations, Expenses ‐ Bristol‐Myers Squibb; Pfizer. Anis A Hamid: Consulting/Honoraria from Bayer. Margaret Ottaviano: Speaker's Bureau from Pharmamar, Travel from Pharmamar, Novartis, Bristol‐Myers Squibb. Alison Reid: Honoraria from Janssen, Astellas (family); Consulting from Janssen, Veridex (family), Roche (family), Astellas(family), Pfizer (family), Novartis (family), Millenium (family), Abbott (family), Essai (family), Bayer (family); Speaker's Bureau from Janssen (family), Astellas (family), Takeda (family), Sanofi‐Aventis (family), Roche (family). Daniel YC Heng: Consulting or Advisory Role: Pfizer, Novartis, Bristol‐Myers Squibb, Janssen, Astellas Pharma. Research Funding: Pfizer (Inst), Novartis (Inst), Exelixis (Inst), Bristol‐Myers Squibb (Inst). Philippe L Bedard: Research Support: Sanofi (Inst), Bristol‐Myers Squibb (Inst), Astra Zeneca (Inst), Genetech/Roche (Inst), Servier (Inst), GlaxoSmithKline (Inst), Novartis (Inst), SignalChem (Inst), PTC Therapeutics (Inst), Nektar (Inst), Merk (Inst), Seattle Genetics (Inst), Mersana (Inst), Immunomedics (Inst). Christoph Seidel, Christian Fankhauser, Thomas Hermanns, Alexey Rumyantsev, Aude Fléchon, Edmond Michael Kwan, Tina Cheng, Giovannella Palmieri, and Robert Kitson: Nil.

## AUTHOR CONTRIBUTIONS

Ben Tran: Conceptualization, data curation, formal analysis, investigation, methodology, project administration, resources, writing—original draft. Jose M Ruiz‐Morales, Enrique Gonzalez‐Billalabeitia, Anna Patrikidou, Christoph Seidel, Carsten Bokemeyer, Christian Fankhauser, Thomas Hermanns, Alexey Rumyantsev, Alexey Tryakin, Margarida Brito, Aude Fléchon, Giovannella Palmieri, Robert Kitson, Alison Reid, and Daniel YC Heng: Data curation, investigation, writing – review and editing. Eitan Amir, Tina Cheng, Daniel Castellano, Xavier Garcia del Muro, Anis A Hamid, Margaret Ottaviano: Formal analysis investigation, writing—review and editing. Edmond Michael Kwan: Data curation, investigation, writing—review and editing Data curation, investigation, writing – review and editing. Philippe L Bedard: Conceptualization, investigation, methodology, supervision, writing—review and editing.
