# Supplementary material for: Large retroperitoneal lymphadenopathy and increased risk of venous thromboembolism in patients receiving first‐line chemotherapy for metastatic germ cell tumors: A study by the global germ cell cancer group (G3)
Source: Cancer Med. 2019 Nov 12;9(1):116–24. doi: 10.1002/cam4.2674 (PMC6943085; doi:10.1002/cam4.2674)
Supplement: Supplementary file 1 [file CAM4-9-116-s001.docx]

**Supplemental Figure A: ROC Analysis determining optimal RPLN diameter in predicting for VTE**

AUC for RPLN >3.5cm is 0.632 (p<0.0001), with sensitivity of 66% and specificity of 63% for VTE

| **Cut-off** | **AUROC** | ***P-value*** |
| --- | --- | --- |
| 2.5cm | 0.613 | *<0.001* |
| 3cm | 0.623 | *<0.001* |
| **3.5cm** | **0.632** | ***<0.001*** |
| 4cm | 0.618 | *<0.001* |
| 4.5cm | 0.608 | *<0.001* |
| 5cm | 0.603 | *<0.001* |
| 6cm | 0.603 | *<0.001* |
| 7cm | 0.600 | *<0.001* |
| 8cm | 0.586 | *0.002* |
| 10cm | 0.561 | *0.03* |

**Supplemental Table B: Bleeding events**

|  | **Entire Cohort**  ***N=1,135*** |
| --- | --- |
| Total number of bleeding events | 17 (1.5%) |
| Bleeding sites  Gastrointestinal  Epistaxis  Brain  Disease-related  Other (Psoas, Bladder, Postoperative)  Unknown | 3  4  3  3  3  1 |
| Bleeding occurring on prophylactic anticoagulation | 0 |
| Bleeding occurring on therapeutic anticoagulation | 2 |
| Intervention (radiological, endoscopic, surgical) required for bleeding | 8 |
| Blood transfusion | 7 |
| Hospitalization required for bleeding | 9 |
| Deaths due to bleeding | 2 |

**Supplemental Table C: Cox proportional hazards model for survival**

|  | **Univariable** | | | **Multivariable** | | |
| --- | --- | --- | --- | --- | --- | --- |
| **Variable** | **HR** | **95% CI** | ***P*** | **HR** | **95% CI** | ***P*** |
| VTE* | 2.84 | 1.81-4.47 | *<0.001* | 1.51 | 0.93-2.46 | *0.10* |
| RPLN >3.5cm | 2.09 | 1.36-3.21 | *0.001* | 1.08 | 0.69-1.71 | *0.73* |
| IGCCCG Risk  Good  Intermediate  Poor | -  3.26  17.95 | -  1.65-6.46  10.48-30.75 | *-*  *0.001*  *<0.001* | -  2.94  14.23 | -  1.45-5.99  7.94-25.50 | *-*  *0.003*  *<0.001* |

*Time-dependent co-variable
